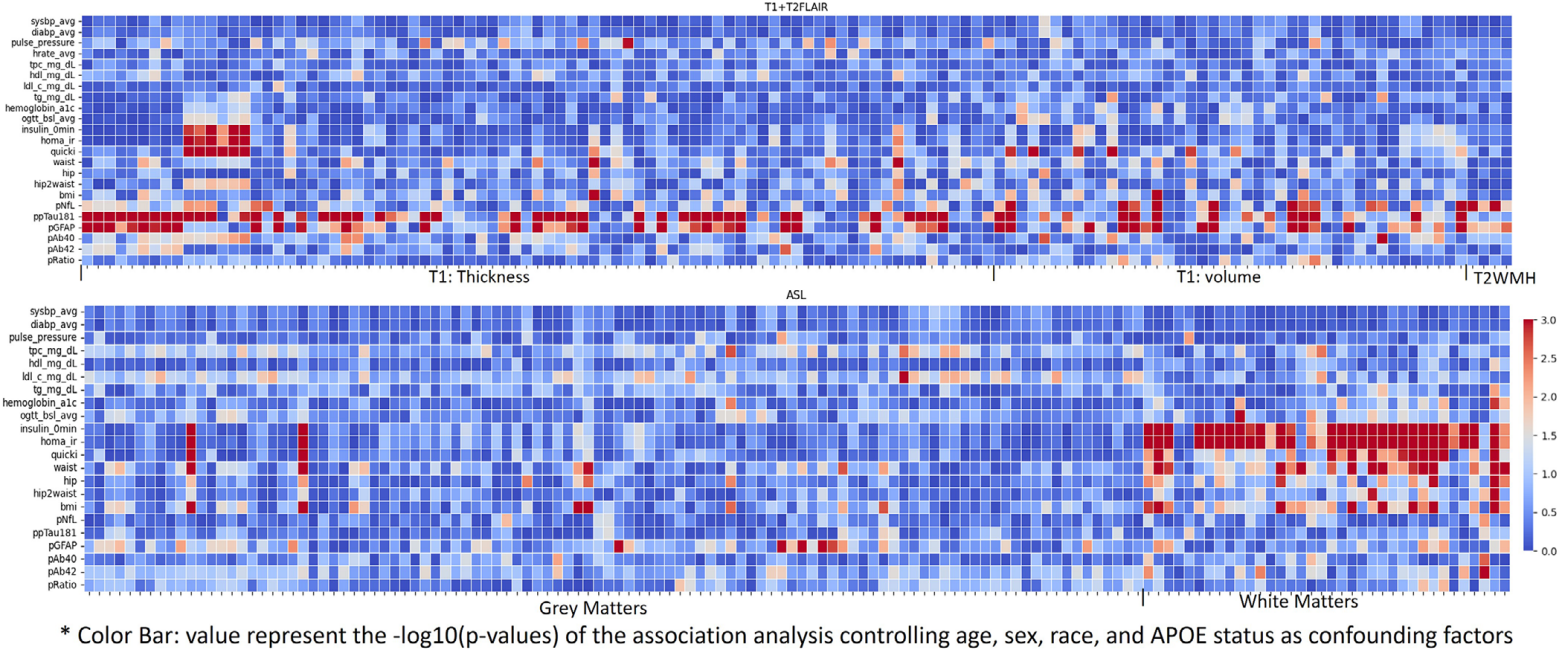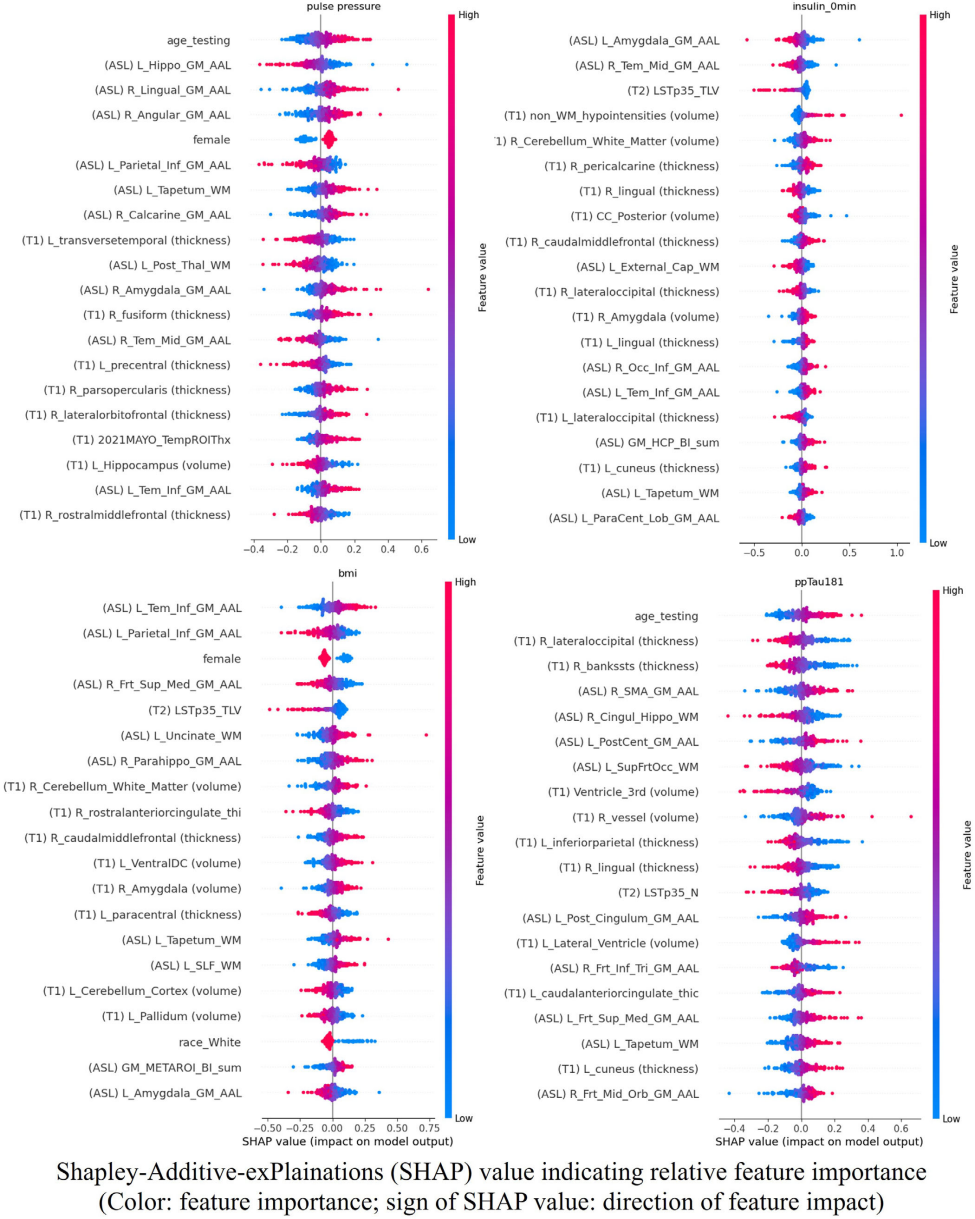# Differential whole‐brain patterns of cardiometabolic risk and blood biomarker using multi‐modal neuroimaging and explainable deep learning

**DOI:** 10.1002/alz.094074

**Published:** 2025-01-09

**Authors:** Da Ma, Samuel N. Lockhart, Timothy M. Hughes, James R. Bateman, Thomas C. Register, Michelle M. Mielke, Metin Nafi Gurcan, Suzanne Craft

**Affiliations:** ^1^ Wake Forest University School of Medicine, Winston‐Salem, NC USA

## Abstract

**Background:**

Cardiometabolic disorders are emerging risk factors for Alzheimer's disease (AD) and AD‐related dementia (ADRD). There is currently insufficient understanding of how different cardiometabolic profiles and blood biomarkers impact different AD‐related brain pathology regionally. This project uses data‐driven approaches and explainable artificial intelligence methods to determine the cardiometabolic and fluid contributions toward AD‐related pathophysiologic patterns in the brain.

**Method:**

A total of 504 participants from the Wake Forest Alzheimer's Disease Research Center (Mean age=70.1, female/male=327/177, cognitive‐comal/mild‐cognitive‐impairment/dementia/other=217/172/45/7, White/Black/Asian/Native=416/81/4/3) had 3T MRI and cardiovascular risk factors (pulse pressure = systolic ‐ diastolic blood pressure), fasting metabolic risk factors (hemoglobin A1c, fasting glucose, insulin, and lipid levels), obesity measures (hip‐to‐waist ratio, and body mass index [BMI]) and plasma biomarker (pTau181, Glial‐fibrillary‐acidic‐protein [GFAP], A Multi‐modal neuroimaging‐based regional brain phenotypes were extracted from T1, T2FLAIR, and ASL imaging sequences to derive structural volume and thickness (FreeSurfer parcellated regions), white matter hyperintensity (WMH) volume, and blood flow information. Regional cerebral blood flow phenotypes were derived from parcellated ASL images registered to the MNI template space using the AAL atlas. Whole‐brain linear association analysis was conducted, followed by a multi‐layer‐perceptron (MLP) deep‐learning‐based non‐linear regression model along with feature importance analysis (Shapley‐Additive‐exPlainations [SHAP]).

**Result:**

Figure 1 shows the heatmap of multi‐modal whole‐brain linear association patterns. Blood biomarkers (pTau181, GFAP) showed an overall strong association with structural data (T1, T2FLAIR), while metabolic factors (esp. fasting insulin and BMI) showed a strong association with ASL (esp. in white matter). The deep‐learning‐based neuroimaging feature importance (SHAP value, Figure 2) further revealed a relatively higher impact of ASL data on various cardiometabolic risk and blood biomarkers than the linear association pattern, indicating potential whole‐brain non‐linear association of cerebral blood flow.

**Conclusion:**

The current study demonstrated differential whole‐brain association patterns across multi‐domain cardiometabolic risk factors. Furthermore, the explainable deep learning approach revealed additional localized brain regions compared to whole‐brain association analysis, especially in gray matter regions from the regional AAL signal. Future studies will incorporate additional neuroimage modalities and derive individual domain‐specific CMR‐associated whole‐brain patterns.